# Supplementary material for: Molecular Marker-Assisted Selection for Frost Tolerance in a Diallel Population of Potato
Source: Cells. 2023 Apr 23;12(9):1226. doi: 10.3390/cells12091226 (PMC10177059; doi:10.3390/cells12091226)
Supplement: Supplementary file 1 [file cells-12-01226-s001.zip › cells-2244290-supplementary.pdf]

cells

MDPI

Supplementary data

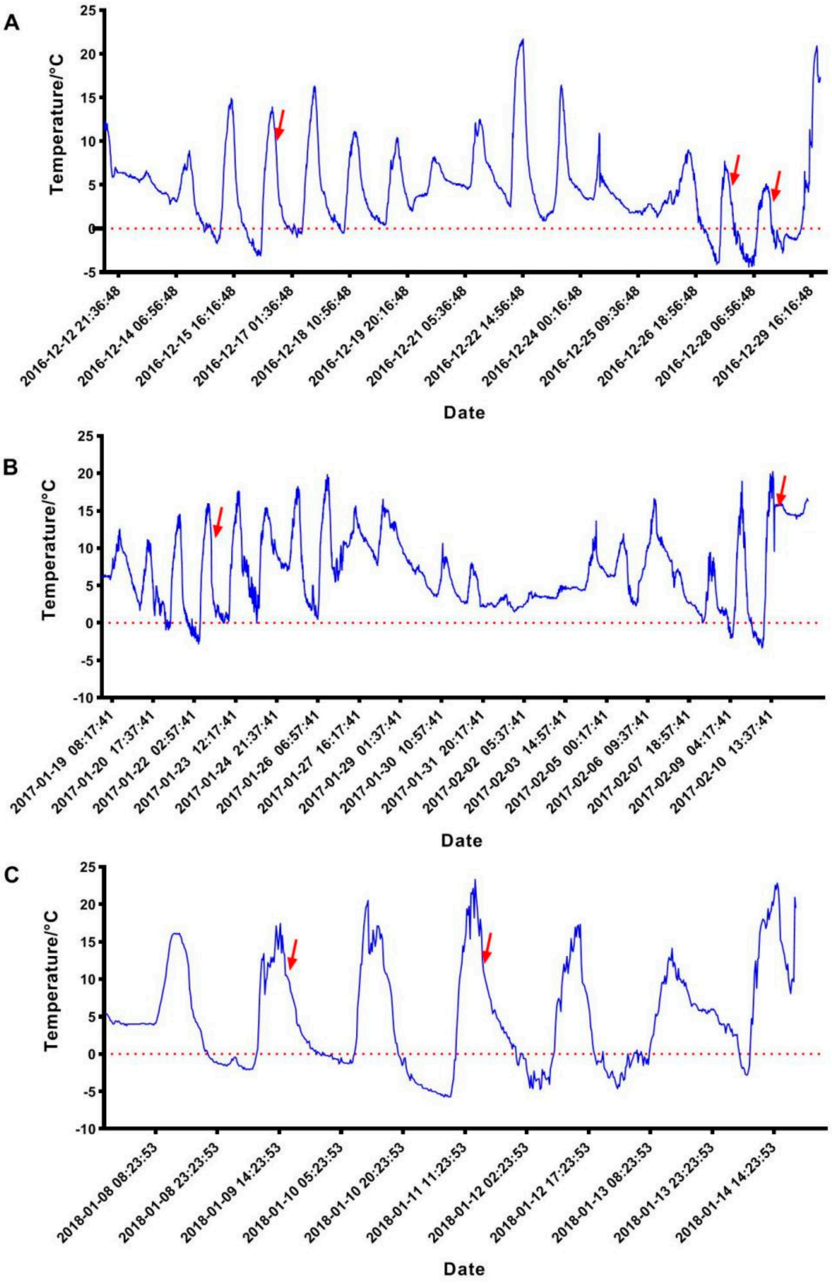

Figure S1. Daily field temperature near the ground for three different field experiments. The temperature was measured 5 cm above the ground at Luoyang in 2016.12 (**A**), Wuhan 2017.01 (**B**), and Wuhan 2018.01 (**C**). Climate data for field experiments were obtained from the temperature recorders (RC-4HC). The date of the assessment of frost tolerance of the parents and F_1 hybrids is marked with arrows.

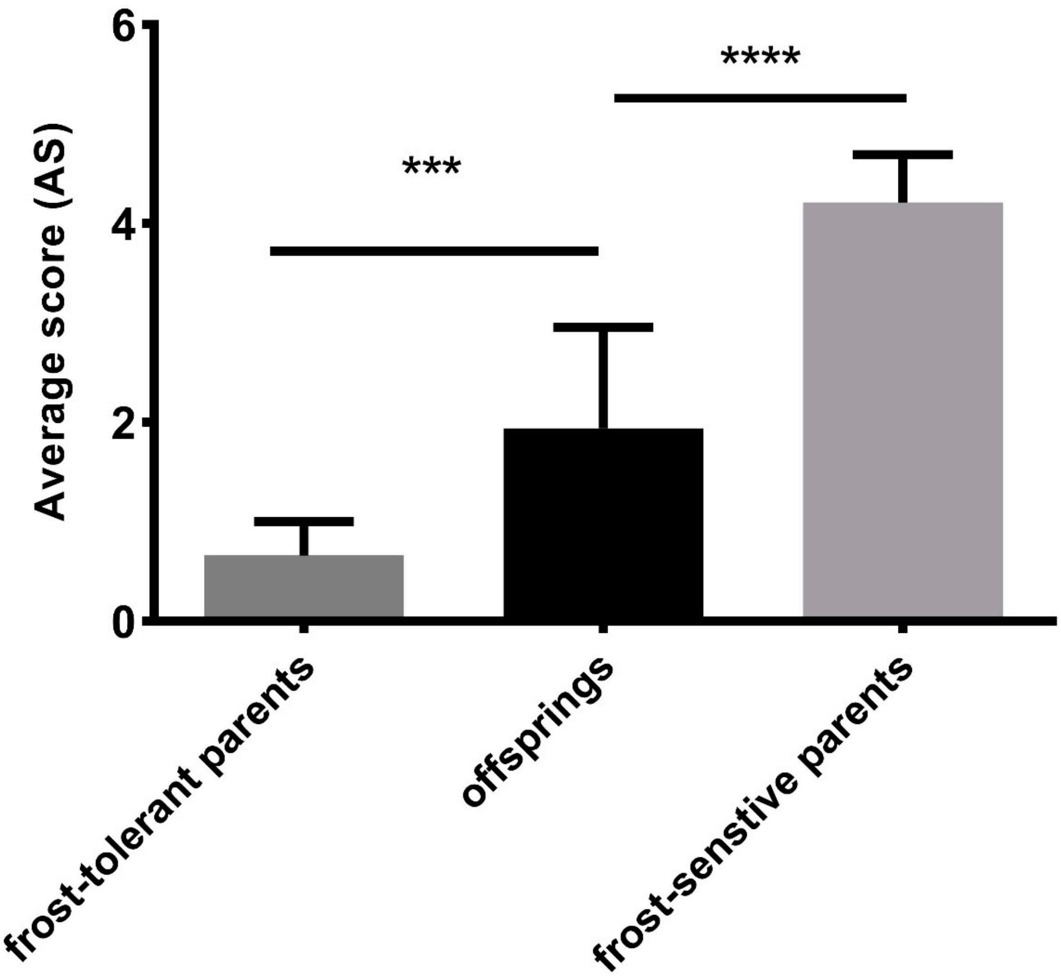

Figure S2. Comparison of frost resistance between multi-parents and progenies. Asterisks indicate significant differences by using Dunnett's multiple comparisons tests (***$P < 0.001$；****$P < 0.0001$).

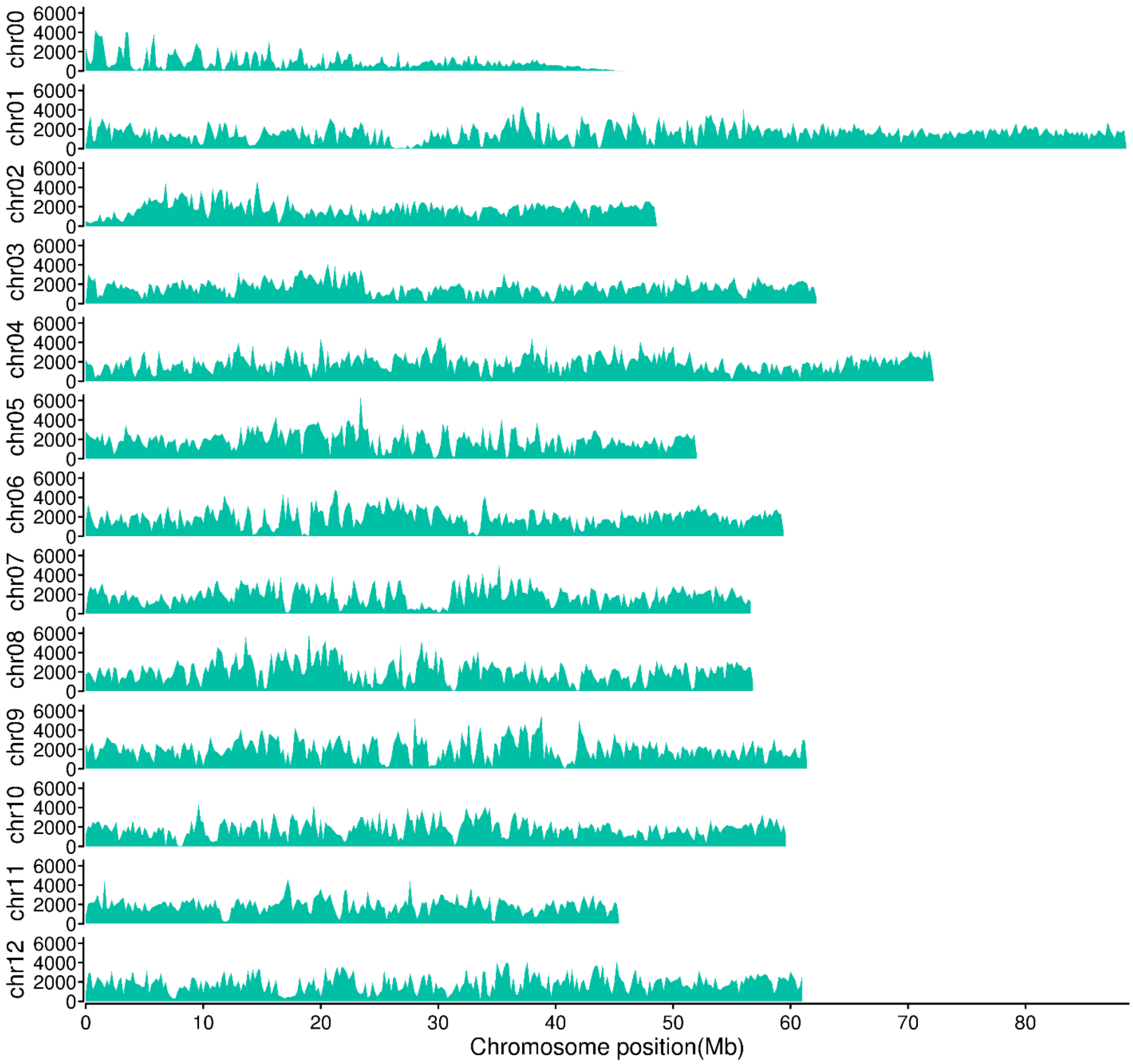

Figure S3. The distribution of available SNP/InDel in the sample on the chromosome 1-12 (with 2 Mb of window size).

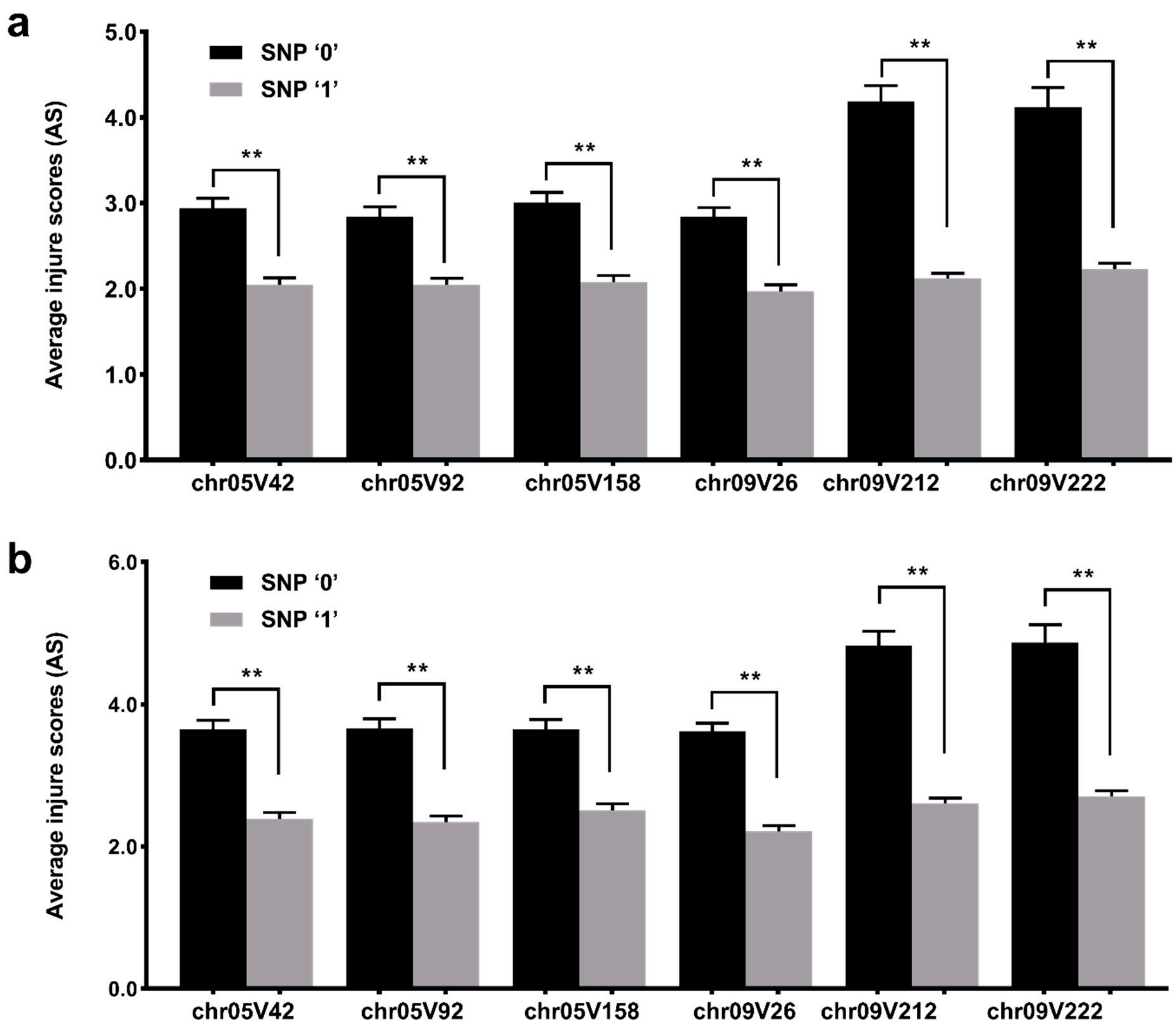

Figure S4. Analysis of the SNP marker individually (genotype '1' or '0') associated with frost tolerance of all progenies in Environment I (**a**) and IV (**b**). Asterisks indicate significant differences using Student's *t*-test (***P*< 0.01).

Table S1. Description of mainly materials used in this study.

Genotype	Ploidy	Species	Accessions	Source
14FT04-25	4x	*S. tuberosum*	BS 214	Sturgeon Bay, Wisconsin
14FT04-44	4x	*S. tuberosum*	BS 215	Sturgeon Bay, Wisconsin
14FT04-63	4x	*S. tuberosum*	BS 216	Sturgeon Bay, Wisconsin
14FT04-71	4x	*S. tuberosum*	BS 217	Sturgeon Bay, Wisconsin
14FT24-10	4x	*S. tuberosum*	BS 218	Sturgeon Bay, Wisconsin
14FT43-25	4x	*S. tuberosum*	BS 219	Sturgeon Bay, Wisconsin
14FT51-08	4x	*S. tuberosum*	BS 220	Sturgeon Bay, Wisconsin
14FT51-03	4x	*S. tuberosum*	BS 221	Sturgeon Bay, Wisconsin
Denali	4x	*S. tuberosum*	AV 9	Sturgeon Bay, Wisconsin
Bora Valley	4x	*S. tuberosum*	634776	Sturgeon Bay, Wisconsin
Pentland Crown	4x	*S. tuberosum*	527906	Sturgeon Bay, Wisconsin
M1	4x	*S. tuberosum*	BS 223	Sturgeon Bay, Wisconsin
M3	4x	*S. tuberosum*	BS 225	Sturgeon Bay, Wisconsin
Plain	2x	*S. tuberosum*		Sturgeon Bay, Wisconsin
RH89-039-16	2x	*S. tuberosum*		Wageningen UR
Huacai 1	4x	*S. tuberosum*		Author

Table S2. Distribution of frost resistance of different cross combinations.

Cross Code	Cross	NO. of off-Springs	The Rank of Frost Resistance [0-2]	(2-4]	(4-6]	Mean of Frost Resistance	Frost Resistance
16FT01	14FT43-25 × 14FT24-10	4	4	0	0	1.14±0.39	R
16FT02	14FT43-25 × 14FT51-08	1	1	0	0	1.49±1.41	R
16FT05	14FT43-25 × RH89-039-16	13	0	11	2	3.64±0.97	MR
16FT06	Bora Valley × 14FT24-10	15	7	7	1	2.48±0.93	MR
16FT07	14FT04-25 × M1	20	20	0	0	1.04±0.41	R
16FT09	14FT04-44 × Pentlond Crown	12	11	1	0	1.16±0.75	R
16FT10	14FT04-44 × Huacai 1	3	0	3	0	3.86±0.65	MR
16FT12	14FT04-63 × 14FT04-63	16	13	3	0	1.66±0.37	R
16FT13	14FT04-63 × 14FT51-08	51	45	6	0	1.54±0.39	R
16FT15	14FT04-63 × M3	10	6	4	0	1.90±0.42	R
16FT18	14FT04-71 × M3	13	8	5	0	1.89±0.70	R
16FT25	14FT24-10 × M3	7	6	1	0	1.26±0.60	R
16FT27	14FT24-10 × RH89-039-16	12	12	0	0	1.11±0.31	R
16FT28	14FT51-08 × 14FT51-08	4	4	0	0	0.88±0.34	R
16FT30	14FT51-08 × M3	4	2	2	0	2.02±0.56	MR
16FT31	14FT51-08 × Plain	1	0	0	1	4.33±1.51	S
16FT32	14FT51-03 × RH89-039-16	8	8	0	0	0.87±0.30	R
16FT33	Denali × 14FT04-25	36	2	18	16	3.64±1.04	MR
16FT34	Denali × 14FT04-44	22	8	14	0	2.46±0.72	MR
16FT35	Denali × 14FT04-63	6	3	3	0	2.46±0.74	MR
16FT36	Denali × 14FT04-71	98	55	43	0	1.95±0.62	R
16FT37	Denali × 14FT24-10	26	11	12	3	2.43±1.07	MR
16FT38	Denali × 14FT51-08	24	11	10	3	2.53±1.00	MR

Table S3. The frost tolerance of parents in various environments.

Cross Parents	Environment						
	I	II	III	IV	V	VI	VII
	2017.1.21[c]	2017.2.21[c]	2018.1.9 [c]	2018.1.11 [c]	2016.12.15 [d]	2016.12.27 [d]	2016.12.28 [d]
14FT04-25 [a]	0.00 ± 0.00	0.17 ± 0.41	0.00 ± 0.00	1.20 ± 0.45	0.00 ± 0.00	0.00 ± 0.00	2.40 ± 1.07
14FT04-44 [a]	0.00 ± 0.00	0.50 ± 0.55	0.20 ± 0.45	1.20 ± 0.45	0.00 ± 0.00	0.00 ± 0.00	2.70 ± 0.67
14FT04-63 [a]	0.67 ± 0.82	0.67 ± 0.82	0.20 ± 0.45	1.20 ± 0.45	0.00 ± 0.00	0.30 ± 0.48	2.10 ± 0.99
14FT04-71 [a]	0.33 ± 0.52	1.00 ± 0.63	1.40 ± 0.55	4.20 ± 0.84	0.00 ± 0.00	0.00 ± 0.00	3.00 ± 0.82
14FT24-10 [a]	0.00 ± 0.00	1.17 ± 0.75	0.60 ± 0.55	1.60 ± 0.89	0.00 ± 0.00	0.00 ± 0.00	1.50 ± 0.55
14FT43-25 [a]	0.00 ± 0.00	3.83 ± 0.75	1.20 ± 0.45	4.00 ± 1.00	0.00 ± 0.00	0.00 ± 0.00	2.50 ± 0.55
14FT51-08 [a]	0.33 ± 0.52	3.83 ± 1.47	0.80 ± 0.45	2.40 ± 1.14	0.00 ± 0.00	0.50 ± 0.71	3.90 ± 0.74
14FT51-03 [a]	0.17 ± 0.41	6.00 ± 0.00	0.20 ± 0.45	1.80 ± 0.84	0.00 ± 0.00	0.00 ± 0.00	1.00 ± 0.47
Average *	0.19 ± 0.24	2.15 ± 2.12	0.58 ± 0.52	2.20 ± 1.24	0.00 ± 0.00	0.10 ± 0.20	2.39 ± 0.98
RH89-039-16 [b]	3.60 ± 0.55	6.00 ± 0.00	3.00 ± 1.00	5.00 ± 1.00	0.80 ± 0.84	5.00 ± 0.71	6.00 ± 0.00
Bora Valley [b]	2.75 ± 0.50	6.00 ± 0.00	4.20 ± 1.30	6.00 ± 0.00	0.80 ± 0.45	4.80 ± 1.30	6.00 ± 0.00
Pentland Crown [b]	3.20 ± 1.30	6.00 ± 0.00	4.20 ± 0.45	6.00 ± 0.00	2.00 ± 1.00	4.00 ± 0.71	6.00 ± 0.00
M1 [b]	2.75 ± 0.50	6.00 ± 0.00	3.20 ± 0.84	6.00 ± 0.00	0.60 ± 0.55	4.20 ± 0.84	6.00 ± 0.00
Hua cai 1 [b]	3.60 ± 0.55	6.00 ± 0.00	5.20 ± 0.45	6.00 ± 0.00	1.00 ± 0.71	6.00 ± 0.00	6.00 ± 0.00
M3 [b]	3.25 ± 0.50	6.00 ± 0.00	4.20 ± 0.45	6.00 ± 0.00	0.40 ± 0.55	5.60 ± 0.55	6.00 ± 0.00
Plain [b]	4.00 ± 0.00	6.00 ± 0.00	3.40 ± 0.55	5.80 ± 0.45	1.60 ± 1.14	6.00 ± 0.00	6.00 ± 0.00
Denali [b]	4.80 ± 0.45	6.00 ± 0.00	3.00 ± 0.71	5.80 ± 0.45	0.80 ± 1.30	5.80 ± 0.45	6.00 ± 0.00
Average *	3.49 ± 0.68	6.00 ± 0.00	3.80 ± 0.78	5.18 ± 0.80	1.00 ± 0.82	5.18 ± 0.80	6.00 ± 0.00

[a] indicates the interspecific hybrids; [b] indicates the cultivated potatoes; [c] indicates the Environment at Wuhan (Environment I, II, III, and IV); [d] indicates the Environment at Luoyang (Environment V, VI, and VII).* indicates the frost resistance of 8 cultivated potatoes or 8 interspecific hybrids in the corresponding environments.Values are Means ± SD. Values are means of ≥3 replicates in each environment.

Table S4. The primers used to develop the SNP markers related to frost tolerance.

Primers	Primer_Left_Sequence	Primer_Right_Sequence
chr05V1*	GCCAGAATGTGGCAACCTGT	TGCAATGTTGATCACACTTGTTGT
chr05V7	TCTAAGGAAACATACCGAACGAAGAGAATA	AAGGTAGGAACCATCTTTTTAGCAGTTTAC
chr05V10	ACCGATGATGACGATACAATAGCTGA	CAATTTAGGCAAAAGAGTGAGAGCCA
chr05V13	GCCTTTCTCGCATCGCGT	GCCTTCCCCCTTTGTTTGCC
chr05V16	CACTCCTTTTCGATTCTAACTACTGTAAGC	CACTTACTCGAGCCTTGAAACAAATTACTA
chr05V22	TGATTTCACCTAGTGGTTGTGGTTTAATTT	CGGGTTAATCTAAATTATTGGGTATGTGCT
chr05V27	GGGGACATGTGAAGTGTTTAGAGTTG	TGCACTCCCTTTTGATTTTAGGCTTT
chr05V31	TGAGAATGACCTTTCATCATAGAACCTACA	GGCTGGTATAGGAGAGATTGTTAGAGATAG
chr05V35*	AACCACCTTGGAGGGCCA	GGGTCCGGGAGTTCTACGC
chr05V38	AGAATGATTCCATATTCACACACAATGGTT	CACTAAATGATCATACCTTTACACGTAGCC
chr05V42*	GTGTCTGCGTCTGCTTCATTCA	ATCGGCGGTGGAGCAGTC
chr05V48*	TCCAAGCAACTCAAAAGCGCA	AGGTGAAGGTCCAACGCAAGA
chr05V51*	AACAGAGGAACTAGGTTCCAACTCAA	CTTAGTCATATCCACCCTCCTTAGGC
chr05V56	CCAAAAAGCTGGTCACCTACTCAAAA	CCATTGGCCTTCTACACACTACCTAT
chr05V61*	TAGAAAGACCTTTTTGATCTTGAGTAAGCC	AAGTTCGACAAAAAGTATCTAAAAGCCCTC
chr05V66	TCTACAAGATCTGCTGAGGTGACTTTATTA	CGAGTCTTTGATCTTGATGTTTATGCTTTG
chr05V69	TCGAAGATCTTGACTAGGATTAAGTTTCCA	ATTGCTTATCATCAACACATTAATGACGGA
chr05V77*	ATTGTTAACATTTTGATTTTGAGTGGGAGG	TTTTCTTTTAATACTCCAGGAGATGGGAGA

chr05V81*	ACCATATATTTGGCTTTGCAAGTTGA	GTAACACACGTGTCTGGAAACTGATT
chr05V86*	TCTGACAACAAGAAACACACCTTCAC	AACAAGGTAAACTGAACGCCGTAATC
chr05V92*	TAGGGTTTCTTCTAGTCTTGGGGTATAGTA	AGAACTTTTCCCGAATATTTGCTTTTCCTA
chr05V98*	ACCGGTCCAAAGGATGTACCA	GCACCAGCCTTTCAATTGGC
chr05V100*	TCTTTCAGGTGAGTTCGACTGTGTAT	CTATTGTTTGACGGTGGGTAGGAATG
chr05V104	GTTAGATCACTATTTCACCCTGTAATGACC	GATAGACAGGCTTGAAGTGTTTTCTGTATT
chr05V107*	TCGTTGCTTGATTCTTGACTATGTAAAGTT	ACTAAAATGAGAAAGAACGACATCCTGAAG
chr05V110*	AGTCACACTCAGCCAACTTAAGGA	GCTCGAGGTCCTGGTCGT
chr05V113	CGACTTTGGAGACACTAGGGGA	AGGTGCGAATCAAAATCGTCGG
chr05V118	CCAAAGGGGTAACGAATGAGAGAATG	ATGCAAGGCCAATAAGTTGTGTATCC
chr05V123*	AGCACAAAGAAAATGCAAAATTGCAAAAAT	TAAATGTTGTACGTCTGAAACTAAATGGGG
chr05V128	CATAATTGCGCTCCATAGCAAACTCT	TGTGTTGTATTTGTATAAAGCGAGAGAAA
chr05V132*	CATCCACACCAAAGCATCACCC	TGTGCCTAGTCTATGGTGGCT
chr05V136	TCCGTGGGAATGCAGTGCT	AGGGCAGTCCCTTTAGAGCAG
chr05V141	AGCTACCCAAGTTAGGGTGCAA	GTCCCGCCGGAACCTGAT
chr05V145	TGATGTAGCCAACATGGGTAATGC	TCACTATGAGCCACGTCAGGA
chr05V148*	TGGCCTCGGCCACTCAAG	CCGTTACTTGGGCCCATATCAGA
chr05V152	TGTTGTAACTCAACTGCATCATCTGG	GCCGGGGAAATTTGGCTTTTAGATTA
chr05V154*	GTAGTGTAGGATTCGATTAGAGCAAGGATA	TTCTTCATTCTTTGAACTCACTTTGTTCCT
chr05V158*	GCCCCGTTACAAACCCTGG	TGCTTGCCTCAAATGCAAGTTCA
chr05V162*	ATAATGAGAACAAGGAGATCAAACACATCG	CAAAAGTCATTGTTCAAGTTTTGTTGTTGG
chr05V165*	CAAGACAAATGATATCAGTCGACCATCTTT	AGCATAGATTTGCATAGAATAGCTTGGTTT
chr09V1	ATCTCCGAGTACCTGTCAATAAACCC	AACTTCTGAGCCTGAAGAAGATGAGT
chr09V8	CAGCAGAAATTGAAATAGGCCTGACA	AGAGATTTTACAACACTTGAAACAACGA
chr09V18	TATAAACCCCACACACTAGTCTGCAA	CTTTCATACTCTGACGATCCATTGCC
chr09V22*	TAAACCAAGACATAAGCTTGAAATGGAACT	TGTCAGTTAGAGAATCTCCCACAAAGTTAT
chr09V26*	ACTGGTCTGATACAAACAATTGATCCAAAA	AGAGAAGTTACAACTCTATCAAGGTTGTCT
chr09V31*	TAGCCCATGGTGATAAGTCGTCTAAC	CACTTGTTCAACTTTGCCTGTTTCAC
chr09V35*	CTGAATGGGGTTGTATAACAATGATGAGAA	CACTCTACTATCCAAAGTCGAAAAACTGAG
chr09V42	ACTAAGTTGTCCAAATATTCCCCATCATTG	TCAAACTTACCCCTAGAAGAATTACCATCA
chr09V47*	GCTCACACTCGCCACTGC	GCACATCGATCAATCAAGGACCC
chr09V51*	TTGTCCTCAAGTAAAACTCAAGTTCAGTAG	CAACTTGTATCTTTGACTGCACAATTCATC
chr09V59*	GTCTATGCTATAATGCTTAAGGAAGCAAGG	ATGTTTTGCATCTTATTCTAGACTTGCACT
chr09V67*	GGGTCTGTCCGTGGGTCG	ACCCCCTTTATGCTGCCCT
chr09V73	GGTTCACCAACTCATTTAAGGAGAGC	TTGGTGCTTGAGAAGTAGTCATGAGA
chr09V78	TGCTACCCTTACAAGCACACTATGAT	GGGTAGCTTGAGTTACCAATGTAGGA
chr09V82	GACCGCTGTCCTAGTTTGACATTAAC	TATCCCAACGGGAAAGATACTGTTGT
chr09V86	AATTAGCTCCTGCCAATTACATCAATTACC	GGAAAGAAGCAGCATTACATGTTCAATAAC
chr09V93	TTTTGGTACCCATACTACACTCTACATCTC	TATACAAATAAGAAATCCCTCCCAGAACCT
chr09V97*	CTGTCCATACCTCCCATCTTTAACCA	CACAAAACAAGTGTAAGGAAGGGTGT
chr09V101	TGATTCCCTTATGAACCCATGCAAAC	AGTTTACCCTTCCAAGGTTCCAAGAT
chr09V106	AGAACTGTAACTAATCCATGCCTAGAATGA	CGTTTCGAGTCTTTCCTTAGATGTTTATGT
chr09V115*	GAATAGATTGGCAATACGCCAAGTGA	AAAGGACCAAGGCATCTGAAAGAATG
chr09V123	AGATTCCATGTTTTTAAGACGAATTCCCAT	TCCTTTATGTGAGAAATCCTTTCACATTCG
chr09V127	CTACTGTTCCTTACTCTCCCCAATCTAATG	AAATATTTCAAGTTGGGTTTTCTGCCTTTC
chr09V131*	ATCTTTAAAGCCAATAGAACCAATTAGCCA	GCATTGACCATGATTACGACTTATGTTTTC
chr09V137	CTCACCAACAGACTCTAAATGCTCAAACTA	TCTTGTTTTAGGATTCCAACTCGCATACTC
chr09V143*	GCTATGTGTCTTAGATGGTGCCTTTC	ATTGTGACACCTCTAAAATGGGGTTG
chr09V148*	TGCAACCTTCTATTCATAAGTGTGGT	TGGCATGTGGAGACTATGTTAGACTT
chr09V154*	ATCTTATGGAGAATGTACAACAGGGTATGA	TTTACTTGTTGACAATTTTGGACATTGAGC
chr09V158*	TTGAAACATGAAATGTGCTATACTCAAAGG	GAAAGTTTGAGTGAGAAAAAGGGAGAAATG

chr09V164	ACTTTCGTGTCCCCCTTTTCATTTAC	TCAACACCCCCAACTTAAATTTTGCT
chr09V170*	AGAGAACAAATTTTTGTACCCACAAAGAGA	TGCATTTCTTTCAGCTGTCTTTTTATTTCC
chr09V175	AATTCGGCGACAAACTAAAGAGATCG	ATTGGTTCCCAGAATTCTATCACGGA
chr09V183*	GGTGAAACCAAGTGCGTTGGA	CGGTCAACGGAGCGGCTA
chr09V188*	TGTAGGCCACACCTTATCCCT	GGTCCACTTATGGTGGCGGA
chr09V197	AGCTCCATGGAAGTGGGTCG	ACCTCTTTCCGAAGGCCACA
chr09V205	TTTTAGGCCAAAACGAGTTCCAAAGT	AAAACGTAAGGTCCGTGACCAATTTT
chr09V212*	CCAAGATTATTGAATTGATGGTCTATGGGT	ACAAGTTGGGTTCTTTACTTGATAACGATT
chr09V218*	AGATTCAAGCTCTCAAGGTCAAGTCT	ACCAATTCCCAATCTCAATTCACCAC
chr09V222*	ACATTAAACAACTTGATTGTTGCGCT	TCATGTATCAGCAACTGTGATTGTCT
chr09V229	ATGACAAACACACGATGATTTGACGA	TTTAACCCATAAACTAACAACCCGCC

The primers with bold font were detected at least once between eight frost-tolerant and eight frost-sensitive parents. * indicates the polymorphism SNP markers.

Table S5. Description of sequencing data in this study.

Sequencing Data Characteristic	Resistant Pool	Sensitive Pool	Mean
Reads	90,170,687	102,494,884	96,332,786
Bases	26,169,018,005	29,674,812,714	27,921,915,360
GC (%)	37.10	37.36	37.23
Q20 (%)	97.38	97.45	97.42
Q30 (%)	92.57	92.73	92.65
No. uniquely mapped	64,261,730	74,894,480	69,578,105
Uniquely mapped (%)	71.27%	73.07%	72.17%
No. repeatly mapped	10,559,095	12,511,242	11,535,169
Repeatly mapped (%)	11.71%	12.21%	11.96%
No. unmapped	15,349,862	15,089,162	15,219,512
Unmapped (%)	17.02%	14.72%	15.87%
Coverage (%)	75.61	76.30	75.96
Mean Depth	22.01	24.93	23.47

Table S6. The 22 SNP markers related to frost resistance between frost-resistant and frost-sensitive parents in various environments.

SNP Name	SNP Genotypes	No. of Resistant Parents	No. of Sensitive Parents	Goodness of Fit (%)	Environment						
					I	II	III	IV	V	VI	VII
chr05V42	G/T (1)	7	0	100	−0.881**	−0.825**	−0.873**	−0.889**	−0.928**	−0.905**	−0.927**
	G/G (0)	0	7								
chr05V81	A/C (1)	7	0	100	−0.881**	−0.825**	−0.873**	−0.889**	−0.928**	−0.905**	−0.927**
	A/A (0)	0	7								
chr05V86	C/C (1)	6	0	100	−0.878**	−0.789**	−0.874**	−0.908**	−0.944**	−0.908**	−0.908**
	T/T (0)	0	5								
chr05V92	A/A (1)	6	0	100	−0.876**	−0.838**	−0.871**	−0.884**	−0.917**	−0.895**	−0.939**
	A/G (1)	1	0								
	G/G (0)	0	8								
chr05V98	C/T (1)	7	4	71.43	−0.723**	−0.431	−0.413	−0.287	−0.600*	−0.698**	−0.484
	T/T (0)	0	3								
chr05V100	C/C (1)	7	4	71.43	−0.307	−0.431	−0.586*	−0.597*	−0.462	−0.338	−0.484
	C/T (0)	0	3								
chr05V107	C/T (1)	5	0	90							

	T/T (0)	1	4		−0.826**	−0.932**	−0.631	−0.757*	−0.784**	−0.706*	−0.539
	T/T (1)	3	0								
chr05V123	C/T (1)	3	1	85.71	−0.717**	−.437	−0.564*	−0.543*	−0.688**	−0.590*	−0.613*
	C/C (0)	1	6								
chr05V154	C/G (1)	7	0	92.31	−0.650*	−0.673*	−0.751**	−0.715**	−0.824**	−0.716**	−0.636*
	C/C (0)	1	5								
	G/G (1)	8	1								
chr05V158	A/G (0)	0	4	92.3	−0.825**	−0.692**	−0.802**	−0.741**	−0.882**	−0.828**	−0.770**
	A/A (0)	0	1								
	G/G (1)	4	0								
chr09V26	A/G (1)	4	0	100	−0.876**	−0.837**	−0.874**	−0.884**	−0.930**	−0.892**	−0.927**
	A/A (0)	0	8								
chr09V31	C/C (1)	8	4	75	−0.537*	−0.483	−0.615*	−0.590*	−0.436	−0.627**	−0.535*
	C/T (0)	0	4								
chr09V35	A/A (1)	4	0	75	−0.632**	−0.708**	−0.473	−0.479	−0.537*	−0.643**	−0.401
	A/C (0)	4	8								
chr09V47	A/G (1)	6	0	87.5	−0.566*	−0.571*	−0.648**	−0.685**	−0.720**	−0.633**	−0.718**
	G/G (0)	2	8								
chr09V59	T/T (1)	8	1	93.75	−0.855**	−0.739**	−0.812**	−0.738**	−0.893**	−0.857**	−0.817**
	A/T (0)	0	7								
	A/A (1)	2	0								
chr09V67	A/C (1)	2	0	73.33	−0.635*	−0.701**	−0.457	−0.460	−0.532*	−0.649**	−0.368
	C/C (0)	4	7								
	T/T (1)	7	0								
chr09V97	C/T (1)	1	3	80	−0.738**	−0.565*	−0.486	−0.462	−0.674**	−0.631**	−0.625**
	C/C (0)	0	5								
chr09V170	G/G (1)	8	3	81.25	−0.576*	−0.565*	−0.456	−0.566*	−0.517*	−0.466	−0.625**
	G/T (0)	0	5								
	A/A (1)	5	0								
chr09V188	A/G (1)	1	0	100	−0.863**	−0.835**	−0.865**	−0.881**	−0.899**	−0.870**	−0.952**
	G/G (0)	0	8								
	G/G (1)	7	0								
chr09V212	A/G (1)	1	0	100	−0.876**	−0.837**	−0.874**	−0.884**	−0.930**	−0.892**	−0.927**
	A/A (0)	0	8								
	A/A (1)	1	0								
chr09V218	A/T (1)	2	0	100	−0.875**	−0.837**	−0.873**	−0.886**	−0.917**	−0.895**	−0.939**
	A/G (1)	4	0								
	G/G (0)	0	8								
	A/A (1)	7	0								
chr09V222	A/C (1)	1	2	87.5	−0.735**	−0.649**	−0.536*	−0.599*	−0.690**	−0.647**	−0.718**
	C/C (0)	0	6								

The environment I, II, III, and IV at Wuhan, and Environment V, VI, and VII at Luoyang. *, ** Significant at the 0.05 and 0.01 probability level, respectively; brackets inside '0' and '1' indicate the frost-tolerant and -sensitive SNP type.
